# IGF-I activates caspases 3/7, 8 and 9 but does not induce cell death in colorectal cancer cells

**DOI:** 10.1186/1471-2407-9-158

**Published:** 2009-05-21

**Authors:** Shi Yu Yang, Capucine Bolvin, Kevin M Sales, Barry Fuller, Alexander M Seifalian, Marc C Winslet

**Affiliations:** 1 University College London, Division of Surgery and Interventional Science, Royal Free & University College Medical School, Rowland Hill Street, London, NW3 2PF, UK; 2 Royal Free Hampstead NHS Trust Hospital, London, UK; 3 University College NHS Hospital, London, UK

## Abstract

**Background:**

Colorectal cancer is the third most common cancer in the western world. Chemotherapy is often ineffective to treat the advanced colorectal cancers due to the chemo-resistance. A major contributor to chemo-resistance is tumour-derived inhibition or avoidance of apoptosis. Insulin-like growth factor I (IGF-I) has been known to play a prominent role in colorectal cancer development and progression. The role of IGF-I in cancer cell apoptosis is not completely understood.

**Methods:**

Using three colorectal cancer cell lines and one muscle cell line, associations between IGF-I and activities of caspase 3/7, 8 and 9 have been examined; the role of insulin-like growth factor I receptor (IGF-IR) in the caspase activation has been investigated.

**Results:**

The results show that exogenous IGF-I significantly increases activity of caspases 3/7, 8 and 9 in all cell lines used; blocking IGF-I receptor reduce IGF-I-induced caspase activation. Further studies demonstrate that IGF-I induced caspase activation does not result in cell death. This is the first report to show that while IGF-I activates caspases 3/7, 8 and 9 it does not cause colorectal cancer cell death.

**Conclusion:**

The study suggests that caspase activation is not synonymous with apoptosis and that activation of caspases may not necessarily induce cell death.

## Background

Normal human colon consists of many crypts; each crypt contains several thousand differentiated cells and a small number of stem cells. Stem cells reside at the bottom of the crypts and divide slowly and systemically, whereas differentiated cells divide rapidly and travel to the top of the crypt. Each day a total of approximately 10^10 ^cells are shed into the colon lumen through apoptosis [1]. Apoptosis is therefore crucial for the maintenance of normal colon morphology and function. When programmed cell death does not occur appropriately in the colon, cells that should be eliminated might persist, become neoplastic and subsequently develop into colorectal cancer (CRC).

CRC is the third most common cancer in the western world. Despite advances in the management of this condition, including improved surgical techniques, the use of chemo or radiotherapy and, more recently, the use of screening, the mortality has not changed for decades. At least 40% of patients with colorectal cancer develop metastases; chemotherapy alone or in combination with radiotherapy is usually used as an adjuvant therapy to surgery for the advanced disease [2]. These approaches, however, are not highly effective against disseminated colorectal metastases [3]. The major contributor to the limited effectiveness of treatment is cancer cell resistance to current chemotherapeutic agents. The most important mechanism which contributes to chemo-resistance is the inhibition or avoidance of drug-induced apoptosis.

Caspases play a central role in most apoptotic cell death, however there is evidence indicated that caspases also involve with non-apoptotic function. For example, caspase 3 activity is required for skeletal muscle differentiation [4] and terminal differentiation of HT-29 colon cancer cells is tightly linked to caspase activation [5]. Caspases also play roles in cell motility, migration and some cell enucleation [6].

It has been established that IGF-I regulates intestinal epithelial cell proliferation, differentiation [7] and that IGF signalling plays a prominent role in cancer development and progression [8-10]. Although many studies have shown that IGF-I is an anti-apoptotic protein, the role of IGF-I in cellular apoptosis is not completely understood. Previous studies have shown that IGF-I fully protected HT-29-D4 colon carcinoma cells undergoing apoptosis induced by tumour necrosis factor-α [11]. Disrupting the interaction between IGF-I and its receptor (IGF-IR) with an IGF-I receptor antagonist significantly increased colon cancer cell apoptosis [12]. There were also some observations, however, showing that IGF-I enhances the apoptotic response to anti-Fas antibody in colorectal cancer cells [13], potentiates tumour necrosis factor-α induced apoptosis in adipose-like cells [14], induces apoptosis in mouse fibroblast cells in the medium with low concentration of serum [15], and also in mouse skeletal muscle cells in the presence of tumour necrosis factor-α (TNF-α) [16]. The precise mechanisms by which IGF-I receptors signal to apoptotic pathways are still unclear. Given the fact that the IGF system has become an attractive molecular target for anticancer therapies, it is necessary and important to determine the relationship between IGF-I pathways and apoptotic pathways.

We have examined the effect of IGF-I on the activities of caspase 8, 9 and 3/7 in three colorectal cancer and one skeletal muscle cell lines. Although these cell lines have different origins, tissue types, cell types and molecular features (table 1), exogenous IGF-I increased the activities of caspase 3/7, 8 and 9 in all of these cell lines and it appear that the IGF-I receptor is involved in caspase activation and the caspase activation does not result in cell death.

**Table 1:** Origins, tissue types, cell types and molecular features of cell lines used in the experiment

Cell line	Species	Tissue	Cell Type	APC	P53	TGF-β IIR
HT-29	Human	Colon	Epithelial	mut	mut	wt

HCT-116	Human	Colon	Epithelial-like	wt	wt	mut

SW620	Human	Colon	Epithelial	mut	mut	wt

C_2_C_12_	Mouse	Muscle	Myblast	wt	wt	wt

## Methods

### Reagents

Recombinant human IGF-I was purchased from Peprotech EC Ltd (UK). IGF-IR antibody was purchased from ab-cam (product code: ab16817, Cambridge, UK). Caspase 3/7, Caspase 8 and Caspase 9 activity assay kits and CellTiter-Blue cell viability assay kits were purchased from Promega (Madison, USA). Yo Pro-1 iodide was purchased from Invitrogen (Paisley, UK). Alamar Blue cell proliferation assay kit was purchased from Serotec Ltd (Oxford, UK). General Caspase inhibitor, Z-VAD-FMK was purchased from R & D System (Oxford, UK). General mouse IgG was purchased from Sigma (product number I 5381, Dorset, UK). All cell culture media, serums and antibiotics were purchased from GIBCO (UK).

### Cell lines and culture conditions

The human colon cancer cell lines HT-29, HCT-116, SW620 and mouse skeletal muscle cell line C_2_C_12 _were purchased from European Collection of Cell Cultures (ECACC). HT-29 and HCT-116 cells were cultured in McCoy's 5A medium containing 2 mM glutamine, 10% foetal bovine serum and 1% penicillin and streptomycin. SW620 cells were cultured in L-15 medium containing 2 mM glutamine, 10% foetal bovine serum and 1% penicillin and streptomycin. C_2_C_12 _cells were cultured in DMEM medium containing 10% foetal bovine serum and 1% penicillin and streptomycin. All cells were cultured and maintained at 37°C with 5% CO_2_/95% air and used at passages 3–8 after their receipt from the supplier.

### Cell treatments

#### IGF-I treated cell

All cells were seeded on 96 well plates with appropriate media (HT-29, HCT-116 cells with McCoy's 5A medium, SW620 cells with L-15 medium and C_2_C_12 _cells with DMEM medium) containing 10% foetal bovine serum and 1% penicillin and streptomycin at the density of 2 × 10^4 ^cells/well. After 24 hours of culture, HT-29 cells were treated with different concentrations (0, 1, 10 and 100 ng/ml) of IGF-I (Peprotech EC Ltd, UK) for 24 or 48 hours with serum containing medium (SCM, McCoy's 5A medium containing 10% foetal bovine serum and 1% penicillin and streptomycin) and serum free medium (SFM, McCoy's 5A medium containing 1% penicillin and streptomycin) respectively. HCT-116, SW620 and C_2_C_12 _cells were treated with concentration of 0 and 50 ng/ml of IGF-I for 48 hours with SCM and SFM respectively. After completion of treatment, cells were subjected to assays for apoptosis, cell death and cell viability (see following section).

#### IGF-IR antibody neutralizing IGF-I actions in cancer cells

Cells were seeded onto 96 well plates with appropriate media (HT-29 cells with McCoy's 5A medium and SW620 cells with L-15 medium) containing 10% foetal bovine serum and 1% penicillin and streptomycin at the density of 2 × 10^4 ^cells/well. After 24 hours of culture, cells were incubated with IGF-IR antibody (product code: ab16817, ab-cam, Cambridge, UK) within SCM or SFM medium respectively at 37°C with 5% CO_2_/95% air for 30 minutes. IGF-I was then added to the wells. The final concentration of IGF-IR antibody in the medium was 400 ng/ml and IGF-I concentration was 50 ng/ml. Cells were incubated with IGF-IR antibody and IGF-I for further 48 hours followed by analysis for apoptotic and cell death assays. A general mouse IgG was also included in the experiment to check whether any IGF-IR antibody neutralization of IGF-I action is a specific effect.

### Caspase activity assay

Following treatments cells were subjected to Caspase 3/7, 8, 9 activities measurement with Caspase-Glo assay kit (Promega, Madison USA). Briefly, the plates containing cells were removed from the incubator and allowed to equilibrate to room temperature for 30 minutes. 100 μl of Caspase-Glo reagent was added to each well, the content of well was gently mixed with a plate shaker at 300–500 rpm for 30 seconds. The plate was then incubated at room temperature for 2 hours. The luminescence of each sample was measured in a plate-reading luminometer (Thermo Labsystems) with parameters of 1 minute lag time and 0.5 second/well read time. The experiments were performed in triplicate and repeated on two separately-initiated cultures.

### Establishment and validation of cell death assay for colorectal cancer cell lines (HT-29, SW620 and HCT116)

In order to investigate whether the caspase activation induces cell death, a previously reported cell death assay [13] was employed to establish cell death assessment for HT-29, SW620 and HCT116 cells. Briefly HT-29, SW620 and HCT116 cells were seeded in 96 well plate for 24 hours and then treated with different concentrations (37.5, 70, 150 and 300 μM) of 5-Fluorouracil (5-FU) which have been shown to be able to induce apoptosis in these cell lines [17-19]. After 48 hours treatment, YO PRO-iodide (Invitrogen) was added to each well at a final concentration of 4.0 μM/L and the plates were incubated at 37°C for further 4 hours. The fluorescence was determined on a fluorescent plate reader (Fluoroskan Ascent FL; Labsystems, Helsinki, Finland). For the comparison purpose the cell death index for treated and untreated cells was calculated with the formula:

where Ft and Fc represent the units of fluorescence (RLU) in the treated and the untreated cells respectively. The untreated cell death index would be 1 (Fc/Fc). A cell death index >1 in the treated cells would indicate more cell death in the treated group.

### Cell death assay

Cells were seeded onto 96 well plates with appropriate media (HT-29 and HCT116 cells with McCoy's 5A medium and SW620 cells with L-15 medium) containing 10% foetal bovine serum and 1% penicillin and streptomycin at the density of 2 × 10^4 ^cells/well. After 24 hours of culture, cells were treated with IGF-I at 100 ng/ml concentration for 48 hours and then subjected to the cell death assay using the above methods.

### Cell viability assay

A previously reported cell viability assay [20] was employed to assess the number of viable cell after IGF-I treatment. Briefly cells were seeded onto 96 well plates with appropriate media (HT-29 and HCT116 cells with McCoy's 5A medium and SW620 cells with L-15 medium) containing 10% foetal bovine serum and 1% penicillin and streptomycin at the density of 2 × 10^4 ^cells/well. Following 24 hours of culture, cells were treated with IGF-I at the concentration of 100 ng/ml for 48 hours. After treatment 20 μl of CellTiter-Blue reagent (Promega, Madison USA) was added to each well and mixed for 2 minute on orbital shaker. Cells were further incubated at 37°C with 5% CO_2_/95% air for 4 hours. The fluorescence was measured with excitation at 530 and emission at 620 nm. The CellTiter-Blue cell viability assay provides a fluorometric method for estimating the number of viable cells. Living cells posses the ability to reduce resazurin into resosurfin, which is highly fluorescent. Nonviable cells, due to loss of metabolic capacity, do not reduce the CellTiter-Blue reagent and thus do not generate a fluorescent signal.

### Inhibition of caspases and cell proliferation assay

In order to investigate whether IGF-I-induced caspase activation has any effect on cancer cell proliferation, a general caspase inhibitor, Z-VAD-FMK (R & D System, Oxford, UK) was used to inhibit caspase activity induced by exogenous IGF-I. Briefly cells were seeded onto 96 well plates with appropriate media (HT-29 and HCT116 cells with McCoy's 5A medium and SW620 cells with L-15 medium) containing 10% foetal bovine serum and 1% penicillin and streptomycin at the density of 2 × 10^4 ^cells/well. Following 24 hours of culture, cells were treated with either IGF-I (100 ng/ml) only or IGF-I (100 ng/ml) plus Z-VAD-FMK (100 μM) for 48 hours. After treatment, 20 μl of Alamar blue (Serotec Ltd, Oxford, UK) was added to each well and mixed for 2 minute on orbital shaker. Cells were further incubated at 37°C with 5% CO_2_/95% air for 4 hours. The fluorescence was measured with excitation at 530 and emission at 620 nm. Alamar blue assay is a redox method to measure the proliferation of various human and animal cell lines and has been proposed as an alternative method to MTT assay [21].

### Statistical Analysis

All data have been examined and followed a normal distribution. All results were expressed as mean ± SEM. One way ANOVA (Prism version 4 2004 edition, USA) with multiple comparison test was used. Statistical analysis was perform on n = 6 samples and Bonferroni's Multiple Comparison Test was used after ANOVA. P < 0.05 is considered as significant and indicated as *; P < 0.01 is considered as higher significance and indicated as **. P > 0.05 is considered as not significant and marked as NS.

## Results

### IGF-I increased caspases 3/7, 8 and 9 activities in colorectal cancer cells

**Figure 1:**
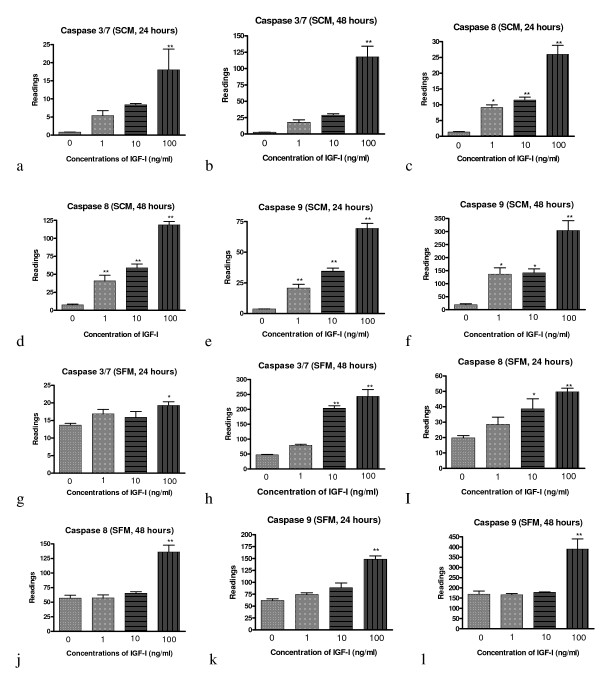
**IGF-I activate caspases 3/7, 8 and 9 in HT-29 cells. ** IGF-I activate caspases 3/7 (a, b, g, h), 8 (c, d, i, j) and 9 (e, f, k, l) in HT-29 cells in serum containing (SCM, a-f) and serum free (SFM, g-l) media following the treatment with different concentrations of IGF-I (1, 10, and 100 ng/ml) for 24 and 48 hours. Significance value: * P <0.05; ** P <0.01 compared to untreated cells.

To assess the effect of IGF-I on the activities of the main caspases (caspase 3/7, 8 and 9) in colorectal cancer cells, we treated the human HT-29 cell line with different concentrations of recombinant human IGF-I for 24 and 48 hours. The caspase activity was evaluated with cell-based homogeneous caspase-glo assay kit in serum containing medium (SCM, figure 1a–f) and serum free medium (SFM, figure 1g–l). The results shown that after treatment with IGF-I for 24 and 48 hours, HT-29 cellular caspase 3/7 activities increased in both SCM and SFM conditions in a dose dependent manner (figures 1a, b, g and 1h). It has been recognized that caspase 3/7 is activated by caspases 8 or 9, therefore these two caspases activity was also measured using the same methods under the same conditions (SCM and SFM). The results demonstrate that after treatment with IGF-I, the activity of the caspase 8 (figures 1c, d, i and 1j) and 9 (figure 1e, f, k and 1l) also increased in SCM and SFM conditions in a dose dependant manner. Caspase 8 activation is the step immediately following death-inducing signalling complex (DISC) formation. The latter is the part of the extrinsic apoptosis pathway which involves death ligands bound to cell surface death receptors. Caspase 9 activation is a part of the intrinsic apoptosis pathway, which involves cytochrome C release from the mitochondria [22]. It is interesting to note that although IGF-I increased in all three caspases (3/7, 8 and 9) activities in both SCM and SFM conditions, the level of these caspases activity in SFM conditions (figure 1g–l) is considerably higher than those in SCM conditions (figure 1a–f). This finding is consistent with a previous report which demonstrated that serum deprivation induced apoptosis [14]. Collectively these data suggested that IGF-I treatment increases human colon cancer HT-29 cell caspase 3/7, 8 and 9 activity under both SCM and SFM conditions.

**Figure 2:**
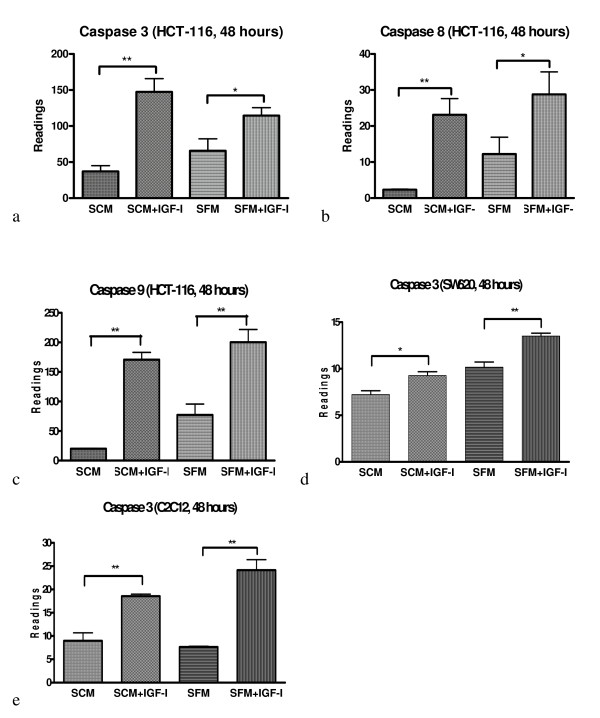
**IGF-I activate caspases 3/7, 8 and 9 in HCT116, SW620 and C2C12 cells**  IGF-I activate caspase 3/7 (a), 8(b) and 9 (c) in serum containing media (SCM) and serum free medium (SFM) in HCT 116 cells following the treatment with IGF-I (50 ng/ml) for 48 hours. IGF-I activate caspase 3/7 in SW620 cells (d) and C_2_C_12_ cells (e) in serum containing media (SCM) and serum free media (SFM) for 48 hours.  Significance value: * P <0.05; ** P <0.01 compared to untreated cells.

To gain further insight into the IGF-I induced caspase activation with other cells; we extended the experiments to SW620 and HCT-116 colorectal tumour cells and C_2_C_12 _myoblast cells. The results demonstrate that addition of IGF-I also significantly increased activity of caspase 3/7 (figure 2a), 8 (figure 2b) and 9 (figure 2c) in HCT-116 CRC cells in both SCM and SFM conditions. For SW620 and C_2_C_12 _cells inclusion of IGF-I in both serum containing and serum free medium also significantly increased caspase 3/7 activities (figure 2d and 2e). Although the cellular properties (including species, tissue type, cell type and gene mutations) in four cell lines used are heterogeneous (table 1), IGF-I universally increased caspase activities in all four cell lines.

### IGF-I activate caspases 3/7, 8 and 9 in colon cancer cells via IGF type 1 receptor

**Figure 3:**
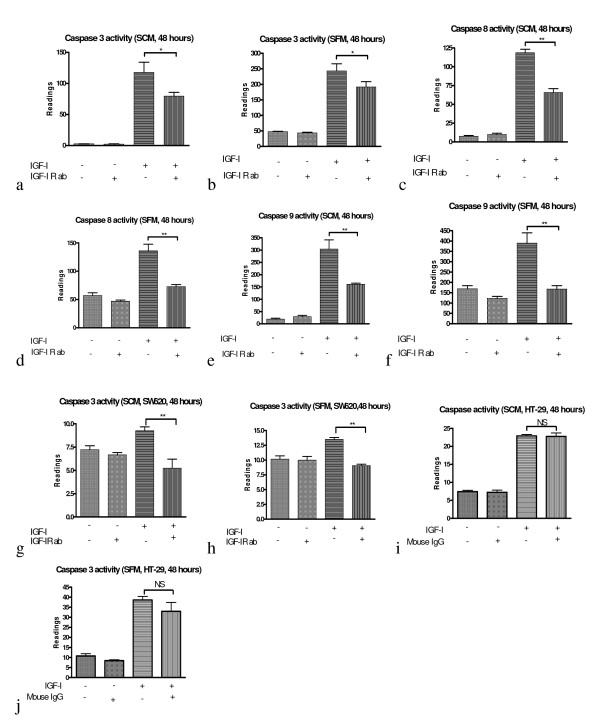
**IGF-I -induced caspases activation is neutralised by anti-IGF-IR antibody**  IGF-I-induced activation of caspases 3/7 (a, b), 8 (c, d) and 9 (e, f) in HT-29 cells and caspase 3/7 in SW620 cells (g, h) is neutralised by anti-IGF type 1 receptor antibody (IGF-IR ab). Cells were pre-incubated with (+) or without (-) IGF-IR ab (400 ng/ml) for 30 minutes and then treated with (+) or without (-) IGF-I (50 ng/ml) for 48 hours. The cellular caspase 3/7, 8 and 9 activities were analysed with Caspase-Glo assay kit (Promega, Madison USA). To check whether any IGF-IR antibody neutralization of IGF-I action is a specific effect a general mouse IgG was included (i, j). For mouse IgG control experiment cells were pre-incubated with (+) or without (-) mouse IgG (400 ng/ml) for 30 minutes. The caspase 3/7 activity was analysed with the same method as IGF-IR ab. Significance value: * P <0.05; ** P <0.01. SCM-serum containing media; SFM-serum free media.

To elucidate whether IGF-I activation of caspases 3/7, 8 and 9 is due to the interaction between IGF-I and its receptors, a neutralised anti-IGF type 1 receptor antibody (IGF-IR ab) was used to interfere with the binding of IGF-I to its receptors in cells. The results show that after 48 hours of IGF-I treatment the activity of caspases 3/7, 8 and 9 in HT-29 cells significantly increased in both SCM (figure 3a, c and 3e) and SFM (figure 3b, d and 3f) mediums. When IGF-IR ab was included in the IGF-I treated cells, the caspase 3/7 activities significantly decreased (P < 0.05) in both SCM (figure 3a) and SFM (figure 3b) mediums compared to IGF-I treated groups, while the caspase 8 and 9 activities significantly decreased (P < 0.01) in both SCM (figure 3c and 3e) and SFM (figure 3d and 3f). All these data show that the caspase 3/7, 8 and 9 activation by IGF-I can be inhibited by interfering in the binding between IGF-I and its receptors and indicate that IGF-I activates caspases 3/7, 8 and 9 in HT-29 cells via interaction between IGF-I and its receptors. In SCM medium (figure 3a, c and 3e) IGF-IR ab did not affect caspase 3/7, 8 or 9 activity if the medium did not include exogenous IGF-I compared to untreated groups. However, in SFM medium the caspase 8 (figure 3d) and 9 (figure 3f) activities tend to be lower in IGF-IR ab treated group compared to untreated groups, although these differences did not reach a statistically significant level. The same effect of blocking the IGF-I receptor with IGF-I R ab was also shown in SW620 cells (figure 3g and 3h). When IGF-IR ab was included in SW620 cells, the caspase 3/7 activities significantly decreased (P < 0.01) in both SCM (figure 3g) and SFM (figure 3h) media compared to IGF-I treated groups. In order to check whether IGF-IR antibody neutralization of IGF-I action is a specific effect; a general mouse IgG was included in the experiment. The results (figure 3i and 3j) showed that general mouse IgG was not able to inhibit IGF-I induced caspase activation. This indicates that IGF-IR antibody effect is a specific action.

### Establishment of cell death assay for colorectal cancer cell lines (HT-29, SW620 and HCT116)

**Figure 4:**
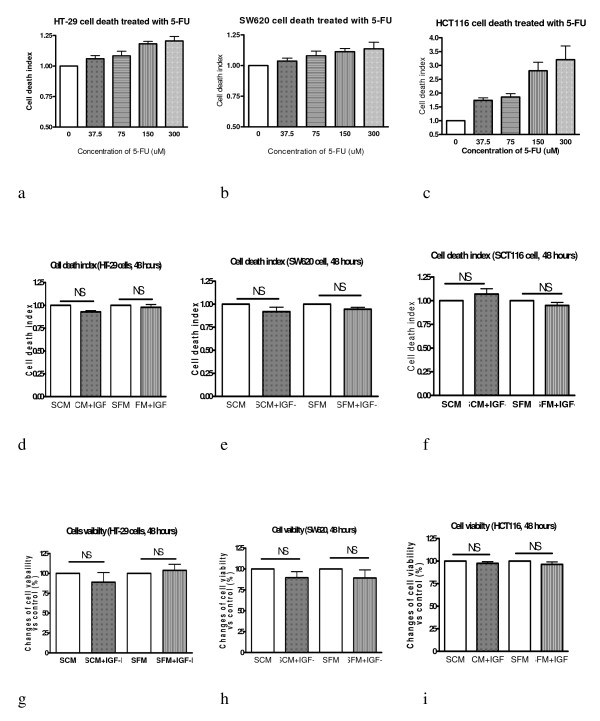
**Exogenous IGF-I dose nod change cell death and viability in HT-29, SW620 and HCT116 cells**. HT-29 (a), SW620 (b) and HCT116 (c) cells were treated with different concentrations of 5-Fluorouracil (5-FU) (0, 37.5, 75, 150 and 300 μM) for 48 hours. The cell death index was determined by a previously reported method  [13]. HT-29 (d), SW620 (e) and HCT116 (f) cells were treated with IGF-I (100 ng/ml) for 48 hours and cell death index was determined with the same method. HT-29 (g), SW620 (h) and HCT116 (i) cells were treated with IGF-I (100 ng/ml) for 48 hours and cell viability was determined by a previously reported cell proliferation assay [20].

After treatment with different concentration of 5-FU, death indexes of HT-29, SW620 and HCT116 cells are shown in figure 4a, b and 4c respectively. Although all three cell lines' death index increased in a dose responsive manner along with the increases of 5-FU concentrations, it is interesting to note that the three colorectal cell lines have different sensitivities to the treatments of 5-FU. While HCT116 cells have the most sensitive responses to 5-FU treatment (figure 4c), HT-29 and SW620 cells only show modest response (figure a and b). These different responses may be due to the divergent genetic background of these cells. HCT116 cell is a p53 wild type of colorectal cancer cell line while HT-29 and SW620 cells contain mutated p53 genes (table 1). Both pre-clinical and clinical studies have shown that disruption of p53 function contributes to 5-FU resistance[23,24]. Never the less, the results have indicated that the death assay can truly represent the actual cell death in each treatment. This death assay method has been successfully used to determine the apoptotic cell death for HT-29 D4, HCT116 and SW620 cells [13]. It is therefore suitable for assessment of cell death in HT-29, SW620 HCT116 cells.

### IGF-I activation of caspases 3/7, 8 and 9 in colon cancer cells does not induce cell death

To further examine whether IGF-I activation of caspases 3/7, 8 and 9 increases apoptosis in colon cancer cells, HT-29, SW620 and HCT116 cells were treated with IGF-I (100 ng/ml) in SCM and SFM conditions respectively for 48 hours. The cell death index was then determined using our established method. The results are shown in figure 4d, e and 4f. It can be seen that cell death is not influenced by exogenous IGF-I at a 100 ng/ml concentration for all three cell lines in SCM and SFM conditions, even though this concentration of IGF-I significantly increase caspase 3/7, 8 and 9 activities in these cell lines (figure 1 and 2). To further confirm that cell death is not induced by caspase activation a previously reported cell viability assessment [12] was used to examine cell survival after treatment with exogenous IGF-I. The results are shown in figure 4g, h and 4i. It can again be seen that cell survival is not significantly different between IGF-I treated and untreated cells in SCM and SFM conditions. These results indicate that IGF-I induced caspase activation alone is not able to be transformed to the ultimate death signal in these colon cancer cells.

### Inhibition of caspase activation induced by IGF-I does not affect cell proliferation

**Figure 5:**
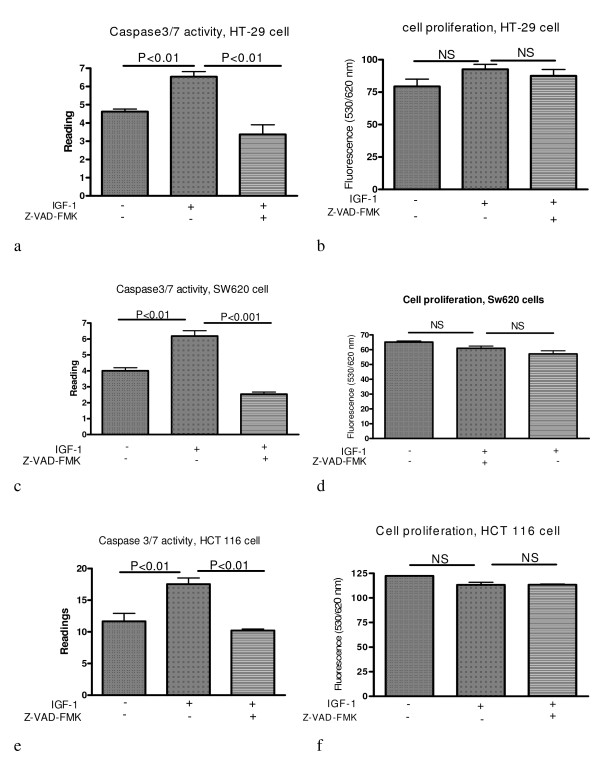
**Inhibition of IGF-I-induced caspases activation does not affect colorectal cancer cell proliferation**. The IGF-I-induced caspase activation is inhibited by incubation with Z-VAD-FMK (a general caspase inhibitor) for 48 hours. The caspase 3/7 activity was measured with Caspase-Glo assay kit (Promega, Madison USA). Cell proliferation was measured with Alamar blue (Serotec Ltd, Oxford, UK). It was shown that Z-VAD-FMK indeed was able to inhibit caspase 3 activation in HT-29 (a), SW620 (c) and HCT116 cells (e). But the inhibition has no effect on cell proliferation (b, d and f).

To investigate whether IGF-I-induced caspase activation has any effect on cancer cell proliferation, caspase activation induced by IGF-I was inhibited by incubation with Z-VAD-FMK (a general caspase inhibitor). It was shown that Z-VAD-FMK indeed was able to inhibit caspase 3 activation in HT-29 (figure 5a), SW620 (figure 5c) and HCT116 cells (figure 5e). But this inhibition has no effect on these cell lines' proliferation (figure b, d and f). These indicate that IGF-I-induced caspase activation has no effect on these cancer cells' proliferation.

## Discussion

IGF-I signalling through the IGF-IR plays an important role in cellular transformation, proliferation and apoptosis of tumour cells. It has been well documented that IGF-IR has the function of mitogenicity, transformation and anti-apoptosis in many cell types, both *in vitro *and *in vivo *[25-27]. In addition to the anti-apoptotic action of IGF-I, there have been several studies which demonstrated that IGF-I can also be pro-apoptotic in colon cancer cells [13], skeletal myoblasts [16], preadipocytes [14], fibroblasts [15] and osteosarcoma cells [28]. The mechanisms by which IGF-I enhances apoptosis in these cells are still largely unclear. This study examined the relation of exogenous IGF-I and the activities of caspase 3/7, 8 and 9 in three colorectal cancer cell lines and one skeletal muscle cell line. It was found that exogenous IGF-I can activate caspases 3/7, 8 and 9 in all four cell lines, and that the activation is via the IGF-I receptor. The four cell lines used in the experiments have a variety of cellular properties including cell species, tissue type, cell type and gene mutations (table 1), nevertheless, IGF-I universally increased caspase activities in all of these cell lines, indicating that caspase activation by exogenous IGF-I is a global effect in all these cells.

Mutational analyses of the IGF-IR have shown that mitogenicity and transforming activity of IGF-IR are localized on separate domains [29-31]. Point mutation in the C terminal of IGF-IR abolished its anti-apoptosis and transformation action, but left its mitogenic action intact [29,32]. Although the molecular mechanism related to pro-apoptotic action of IGF-I R remain unclear, expression of the IGF-IR C terminal as a myristylated protein caused massive cell death [33] indicating IGF-IR C terminal plays a critical role in regulating cell apoptosis. It has also been proposed that segregation of IGF-IR in and out of membrane lipid rafts may regulate the pro and anti-apoptotic effects of IGF-I [13]. The finding that IGF-I activation of caspases in colorectal cancer and C2C12 cells can be inhibited by blocking IGF-I binding to IGF-IR receptor in this study shows that IGF-IR indeed is involved in regulation of IGF-I-induced caspase activation. It is interesting to note that in some conditions IGF-I R ab only partially inhibit IGF-I induced caspase activation and inhibition effect is more effective in SFM conditions than in SCM conditions (figure 3). This indicates that there may be another receptor or growth factor which is involved in the caspase activation.

Although it is widely assumed that the apoptotic death of mammalian cells is closely associated with activation of caspases, there is evidence indicating that caspase activation is also involved with processes that are not necessarily related to apoptosis. For examples, caspase 3 activity is involved with skeletal muscle differentiation [4] and when HT-29 cells are induced to the terminal differentiation caspase activation is required [5]. It has been reported that caspase activation may contribute to subtle signalling pathways, some of which may enhance cell survival and proliferation [34]. Our results showed that IGF-I activates caspases 3/7, 8 and 9 in colorectal cancer calls, but did not cause cell death. This indicates that caspase activation induced by exogenous IGF-I may be involved with processes not related to the apoptotic cell death. A further study is currently investigating the role of IGF-I induced caspase activation. These findings raise an important question, i.e. caspase activation is not synonymous with apoptotic death; therefore increased caspase activities can not be regarded as a sole indicator of cell death.

## Conclusion

IGF-I activates caspase 3/7, 8 and 9 in three colon cancer and one skeletal muscle cell lines and IGF-IR is involved in the IGF-I induced caspase activation. IGF-I induced caspase activation is not able to be transformed to death signal; it may be involved with other processes which are not related to apoptosis.

## Abbreviations

IGF-I: Insulin-like growth factor I; IGF-IR: Insulin-like growth factor type 1 receptor; IGF-IR ab: Anti Insulin-like growth factor type 1 receptor antibody; CRC: Colorectal cancer; TNF: Tumour necrosis factor; SCM: Serum-containing media; SFM: Serum-free media.

## Competing interests

The authors declare that they have no competing interests.

## Authors' contributions

SYY and MCW conceived the study. SYY designed the experiments, carried out the study and prepared the manuscript. CB carried out partial experiments. KMS, BF, AMS and MCW participated in the design, reviewed all data, and contribute in the preparation of the manuscript. All authors read and approved the final manuscript.

## Pre-publication history

The pre-publication history for this paper can be accessed here:

http://www.biomedcentral.com/1471-2407/9/158/prepub
